# ELTD1 Activation Induces an Endothelial-EMT Transition to a Myofibroblast Phenotype

**DOI:** 10.3390/ijms222011293

**Published:** 2021-10-19

**Authors:** Helen Sheldon, John Alexander, Esther Bridges, Lucia Moreira, Svetlana Reilly, Koon Hwee Ang, Dian Wang, Salwa Lin, Syed Haider, Alison H. Banham, Adrian L. Harris

**Affiliations:** 1 Cancer Research UK Molecular Oncology Laboratories, University of Oxford, Weatherall Institute of Molecular Medicine, John Radcliffe Hospital, Oxford OX3 9DU, UK; esther.bridges@ndm.ox.ac.uk (E.B.); david.ang1387@gmail.com (K.H.A.); salwa.lin@seh.ox.ac.uk (S.L.); 2 The Breast Cancer Now Toby Robins Research Centre, Division of Breast Cancer Research, The Institute of Cancer Research, London SM2 5NG, UK; john.alexander@icr.ac.uk (J.A.); Syed.Haider@icr.ac.uk (S.H.); 3 Cardiovascular Medicine, RDM John Radcliffe Hospital, Oxford OX3 9DU, UK; lucia.moreira@cardiov.ox.ac.uk (L.M.); Svetlana.reilly@cardiov.ox.ac.uk (S.R.); 4 Nuffield Division of Clinical Laboratory Sciences, Radcliffe Department of Medicine, John Radcliffe Hospital, Oxford OX3 9DU, UK; dian.wang@lmh.ox.ac.uk (D.W.); alison.banham@ndcls.ox.ac.uk (A.H.B.)

**Keywords:** ELTD1, EMT, pro-angiogenic, myofibroblast

## Abstract

ELTD1 is expressed in endothelial and vascular smooth muscle cells and has a role in angiogenesis. It has been classified as an adhesion GPCR, but as yet, no ligand has been identified and its function remains unknown. To establish its role, ELTD1 was overexpressed in endothelial cells. Expression and consequently ligand independent activation of ELTD1 results in endothelial-mesenchymal transistion (EndMT) with a loss of cell-cell contact, formation of stress fibres and mature focal adhesions and an increased expression of smooth muscle actin. The effect was pro-angiogenic, increasing Matrigel network formation and endothelial sprouting. RNA-Seq analysis after the cells had undergone EndMT revealed large increases in chemokines and cytokines involved in regulating immune response. Gene set enrichment analysis of the data identified a number of pathways involved in myofibroblast biology suggesting that the endothelial cells had undergone a type II EMT. This type of EMT is involved in wound repair and is closely associated with inflammation implicating ELTD1 in these processes.

## 1. Introduction

EGF, latrophilin and seven transmembrane domain-containing protein 1 (ELTD1), recently re-designated Adhesion G Protein-Coupled Receptor L4 (ADGRL4), is an orphan adhesion G Protein-Coupled Receptor (GPCR) of the Latrophilin family. It was first identified in 2001 by Nechiporuk et al. and was expressed in cardiomyocytes and smooth muscles during heart development [1]. ELTD1 has a long extracellular N-terminus containing epidermal growth factor (EGF) and calcium-binding EGF domains, these domains are found in other adhesion GPCRs where they mediate cell-cell interactions and cell migration [2]. The extracellular region of ELTD1 also contains a membrane proximal GPCR-Autoproteolysis INducing (GAIN) domain which undergoes autocatalytic processing at a GPCR-proteolytic site (GPS) to give rise to an extracellular domain that is non-covalently bound at the cell surface [3].

Several members of the adhesion GPCR family have been implicated in angiogenesis. GPR124 is highly expressed in the endothelium of the central nervous system (CNS) and is essential for regulating CNS angiogenesis [4]. CD97 stimulates angiogenesis by binding to integrin on endothelial cells [5] and loss of Gpr116 in mice has also been reported to result in a subtle vascular phenotype [6]. ELTD1 expression was identified in normal vasculature [7] and tumour vasculature [8]. We later confirmed this finding by showing greater protein expression in tumour vasculature compared to matched normal tissues in a number of tumour types [8]. ELTD1 has also been reported to be up-regulated in glioblastoma cells as well as vasculature [9,10]. ELTD1 expression is regulated by VEGF [8] and in vitro and zebrafish studies identified ELTD1 as a key regulator of sprouting angiogenesis. More importantly, the growth of human ovarian and colorectal tumour xenografts were substantially inhibited using anti-mouse Eltd1 silencing [8], suggesting ELTD1 is a potential target for anti-angiogeneic therapy. Adhesion GPCRs are a relatively new GPCR subfamily and there is limited information on their functions, especially since most have no known ligands [11]. Increased expression of GPCRs has been successfully used to help deorphanize receptors and characterise their signalling pathways [12]. In the absence of a ligand for ELTD1 we utilised this approach to help establish its role in endothelial cells.

## 2. Results

### 2.1. Expression of coELTD1 in Endothelial Cells

**Figure 1:**
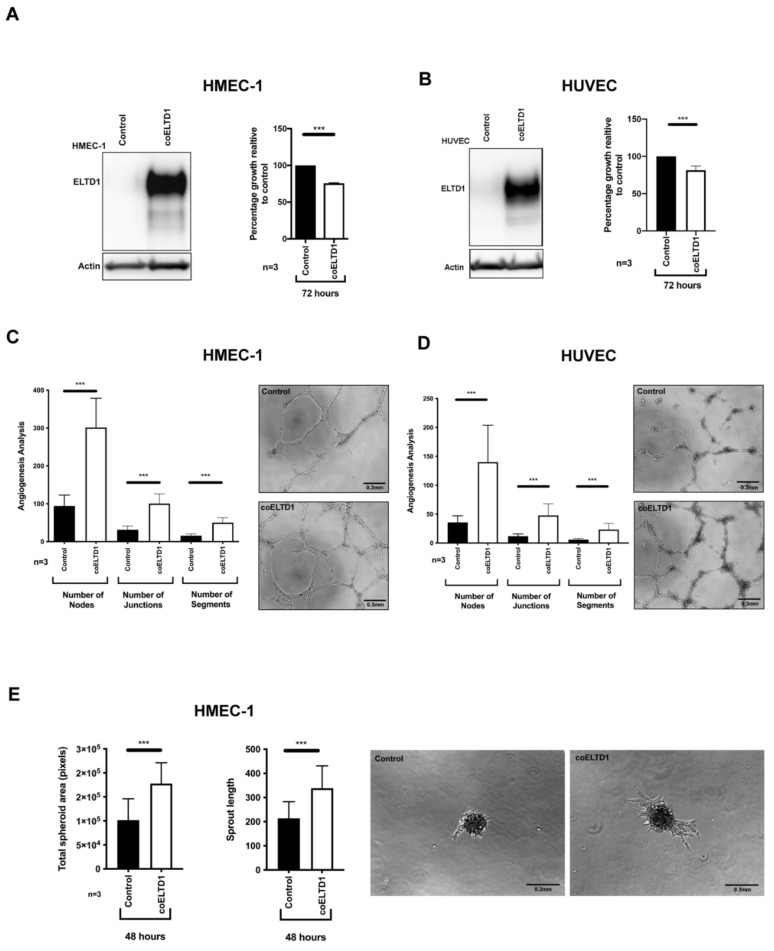
coELTD1 expression is pro-angiogenic. (**A**) Western blot of coELTD1 expression in HMEC-1 and effect of expression on proliferation. (**B**) Western blot of coELTD1 expression in HUVEC and effect of expression on HUVEC proliferation. (**C**) Matrigel assay on coELTD1 expressing HMEC-1 with quantification of network formation. (**D**) Matrigel assay on coELTD1 expressing HUVEC with quantification of network formation. (**E**) Sprouting assay in Matrigel^TM^ of coELTD1 expressing HMEC-1 with quantification of spheroid area and sprout length. Images were taken after 48 h on an AMG Evos XL Core digital microscope (Fisher Scientific, Waltham, MA, USA) at 10× magnification. *** *p* < 0.0005.

Expression of coELTD1 was used to activate the receptor in the absence of a ligand by increasing the proportion of receptors in the active conformation [13]. The endogenous sequence of ELTD1 is poorly expressed therefore the cDNA was codon optimised using the JCat bioinformatics tool (http://www.jcat.de/) (accessed on 31 November 2017) without altering the amino acid coding sequence. coELTD1 expression was confirmed in the stable human microvascular endothelial cell line HMEC-1 and in human umbilical vein endothelial cells (HUVEC) by Western blotting (Figure 1A,B). Expression of coELTD1 reduced cell growth in both cell types by approximately 25% (Figure 1A,B) but increased angiogenic activity as evidenced by increased Matrigel^TM^ network formation (Figure 1C,D) and increased sprouting of HMEC-1 cells (Figure 1E).

**Figure 2:**
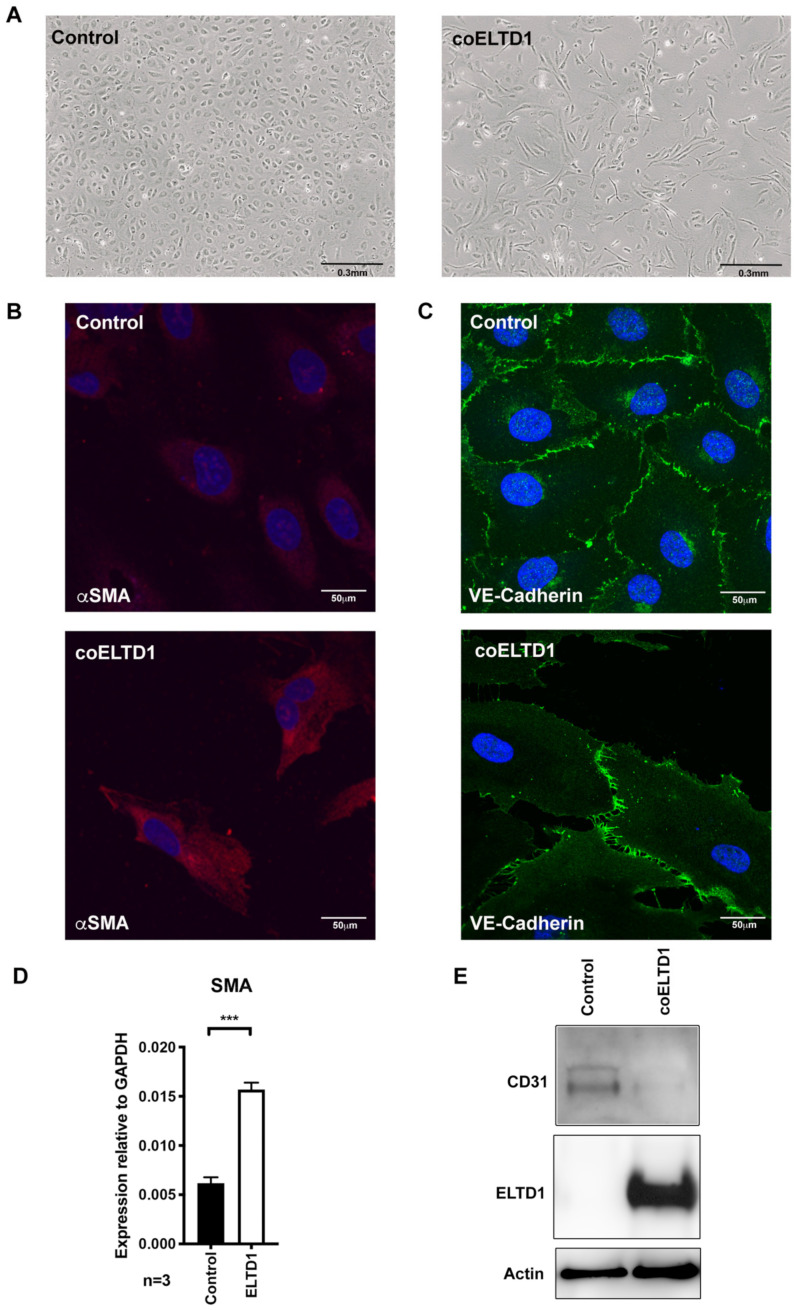
coELTD1 expression induces EndMT. (**A**) Visualization of HUVEC 120 h post infection with control and coELTD1 expressing lentivirus. Images were taken on an AMG Evos XL Core digital microscope (Fisher Scientific, Waltham, MA, USA) at 10× magnification. (**B**) Fluorescent staining of αSMA in control and coELTD1 expressing HUVEC cells at 120 h. (**C**) Fluorescent staining of VE-Cadherin in control and coELTD1 expressing HUVEC cells at 120 h. Images were taken on a Zeiss LSM 880 Confocal Microscope (Zeiss, Oberkochen, Germany) at 63× magnification. (**D**) QPCR of α*SMA* in control and coELTD1 expressing HUVEC. (**E**) Western blot analysis of CD31 expression in control and coELTD1 expressing HUVEC. *** *p* < 0.0005.

Approximately 5 days after infection with a coELTD1-expressing lentivirus, both cell types underwent a form of EndMT, exhibiting an elongated spindle-like morphology (Figure 2A and Control Video S1 Control and Video S2 ELTD1). The cells had an increased expression of alpha smooth muscle actin (αSMA), which is a mesenchymal marker of EMT [14] (Figure 2B,D), loss of intercellular contacts at VE-Cadherin junctions (Figure 2C) and and a decrease in endothelial CD34 expression when compared to infected controls (Figure 2E).

### 2.2. Expression of Tagged coELTD1

**Figure 3:**
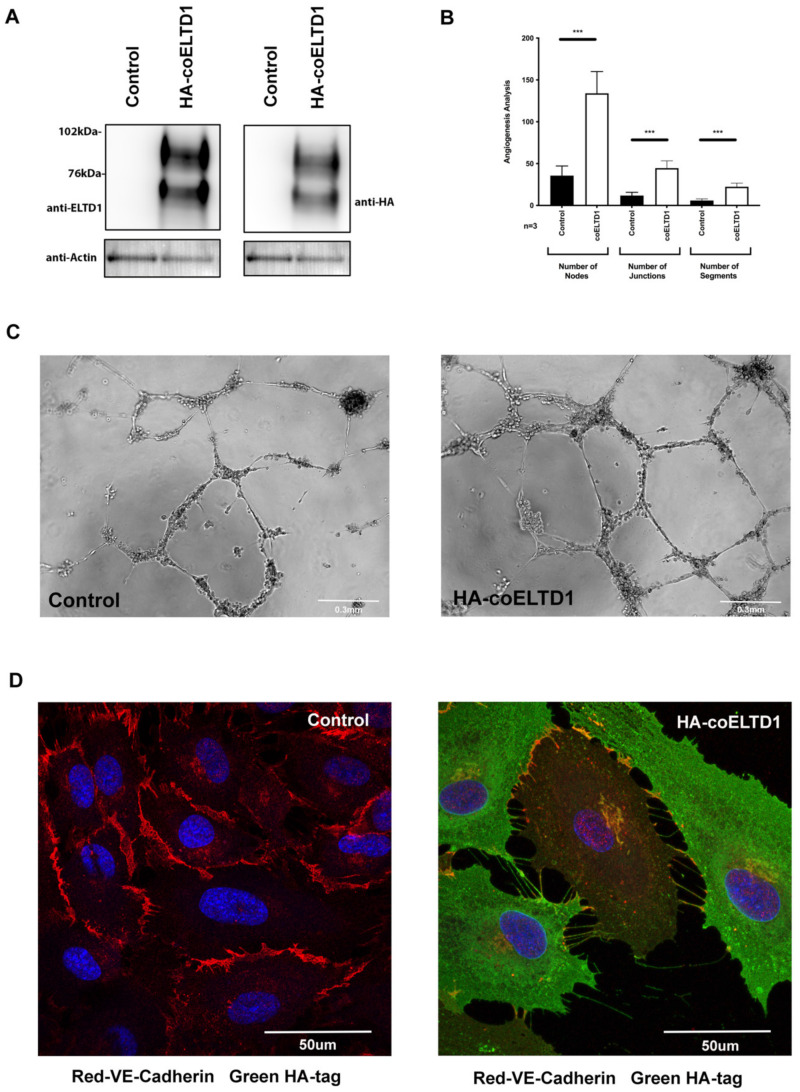
HA and FLAG tagging of coELTD1. (**A**) Western blot of HUVEC cells transduced with control versus HA-tagged coELTD1 expressing lentivirus probed with anti-ELTD1 and anti-HA tag antibodies. (**B**,**C**) Matrigel assay of control versus HA-tagged coELTD1 expressing HUVEC with quantification of network formation. (**D**) Fluorescent staining of VE-Cadherin and HA-tagged coELTD1 in control and HA-coELTD1 expressing HUVEC cells at 120 h. Images were taken on a Zeiss LSM 880 Confocal Microscope (Zeiss) at 63× magnification. *** *p* < 0.0005.

Commercially available anti-ELTD1 antibodies did not work well for immunofluorescence and we had not yet developed in-house antibodies, therefore we tagged the extracellular domain (ECD) with HA. HA-tagging did not affect the processing and glycosylation of the ECD as an antibody raised against the ECD of ELTD1 and the HA-Tag antibody gave the same two bands described previously by our laboratory which correspond to the two glycosylated forms of the ECD [8] (Figure 3A). The increased expression of ELTD1 and HA-tagging did not appear to interfere with protein localisation as immunofluorescent staining of HUVEC cells using an anti-ELTD1 antibody against the ECD gives a similar pattern in control, coELTD1 and HA-tagged coELTD1 expressing cells (Figure S2A,B).

HUVEC cells expressing HA-tagged coELTD1 had the same phenotype as untagged coELTD1 with an increased network formation on Matrigel^TM^ (Figure 3B,C). HA-tagged coELTD1 expressing HUVEC also had a loss of cell-cell contact with HA-tagged coELTD1 localised on stretch points between detached cells anchored by VE-cadherin (Figure 3D).

### 2.3. Confocal Microscopy of coELTD1-Induced Shape Changes

**Figure 4:**
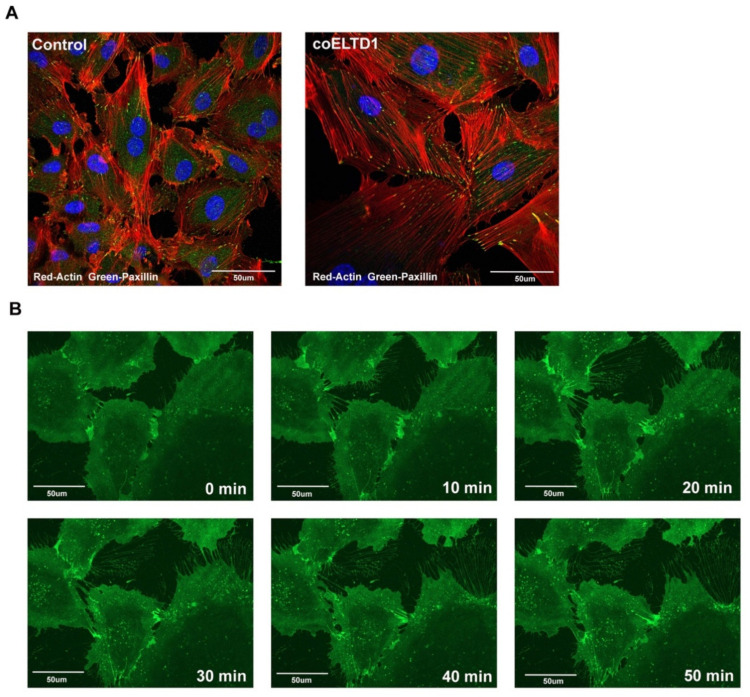

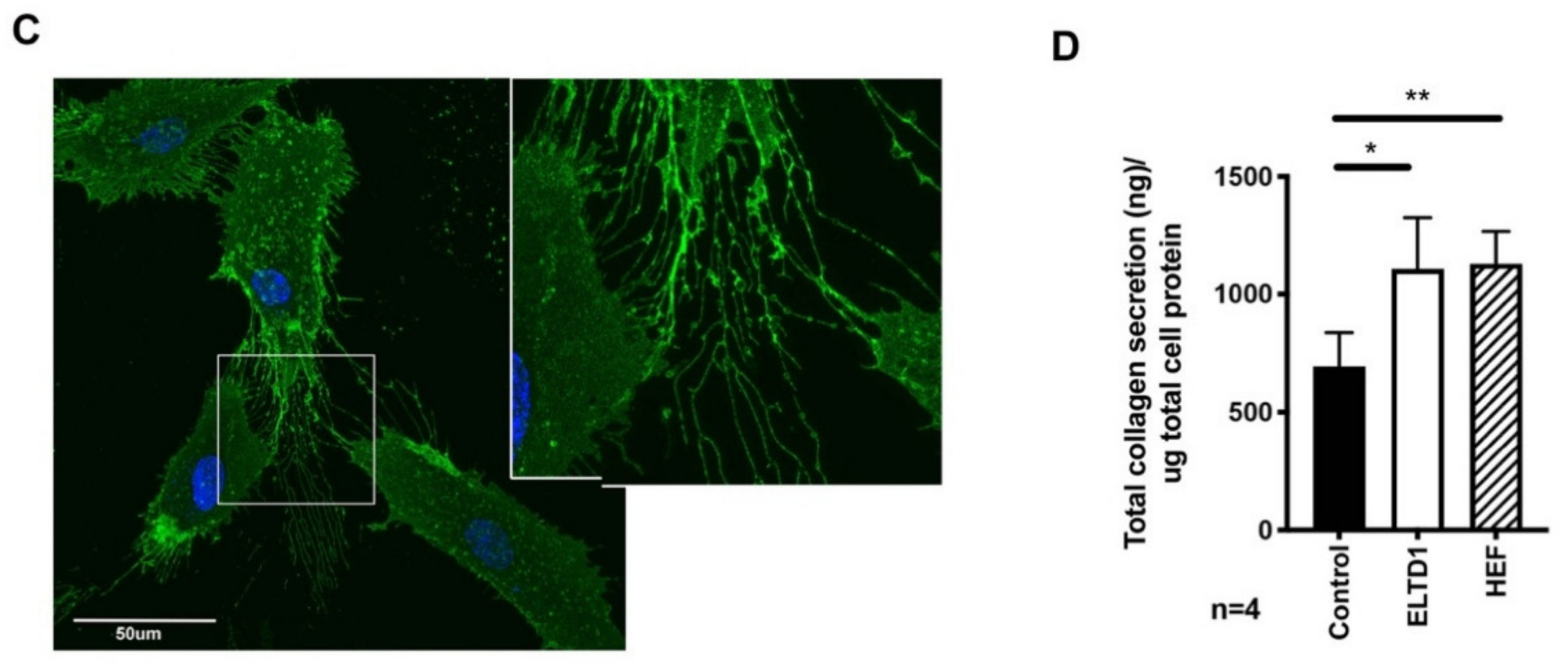
coELTD1 expression induces myofibroblast-like shape changes. (**A**) Fluorescent staining of actin (phalloidin) and paxillin in control and coELTD1 expressing HUVEC. Images were taken on a Zeiss LSM 880 Confocal Microscope (Zeiss) at 40× magnification. (**B**) Still images taken from HA-tagged coELTD1 Video S3 at 10 min intervals. HUVEC cells were infected with HA-tagged coELTD1 and imaged 10 min after addition of HA-Alexa Fluor 488 using a Zeiss Observer spinning disc confocal microscope. (**C**) Membrane staining of non-permeabilized HUVEC cells expressing HA-tagged coELTD1 using anti-HA-Alexa Fluor 488 with zoomed inset of cell tracks. Images were taken on a Zeiss LSM 880 Confocal Microscope (Zeiss) at 63× magnification. (**D**) Collagen assay performed on supernatant collected from HUVEC cells 120 h after infection with control or coELTD1 virus. * *p* < 0.05; ** *p* < 0.005.

coELTD1 expressing HUVECs adopt a myofibroblast-like phenotype. Myofibroblasts are characterised not only by expression of αSMA but also by the formation of stress fibres and large focal adhesions which enable to cells to contract and close wounds during healing [15]. To confirm these myofibroblast like changes, coELTD1 expressing cells were stained with TRITC-phalloidin to visualise actin and paxillin to locate the focal adhesions. coELTD1 expression increased the size of the cells and re-organised the actin into stress fibres with large focal adhesions confirming myofibroblast formation (Figure 4A).

The HA-tagging allowed us to visualise the sub-cellular localisation of ELTD1 by live cell imaging. Live microscopy was performed on HA-tagged coELTD1 expressing HUVEC 10 min after the addition of an Alexa Fluor 488^®^ tagged HA antibody (HA-tagged coELTD1 Video S3 and Figure 4B). HA-tagged coELTD1 was present at the cell membrane and accumulates at points of cell-cell contact when the cells begin to detach. It was also left on cell tracks as the cells migrated, suggesting a strong adhesion to the tissue culture plate surface. Higher magnification of this phenomenon showed extensive ELTD1 positive membrane deposition with circular macroaggregates present, that are thought to be integrin rich [16] (Figure 4C). The mechanism for production of these circular structures is unknown, but suggest vesicle production.

Myofibroblasts also secrete extracellular matrix proteins (ECM) such as collagens, glycoproteins, proteoglycans and elastins to provide structure, cell guidance and a reservoir of growth factors during wound repair [17], therefore collagen secretion was quantified and found to be significantly higher in the supernatant of coELTD1 and HA-tagged coELTD1 expressing cells (Figure 4D).

### 2.4. RNA-Seq of coELTD1 mRNA Expression Profiles

**Figure 5:**
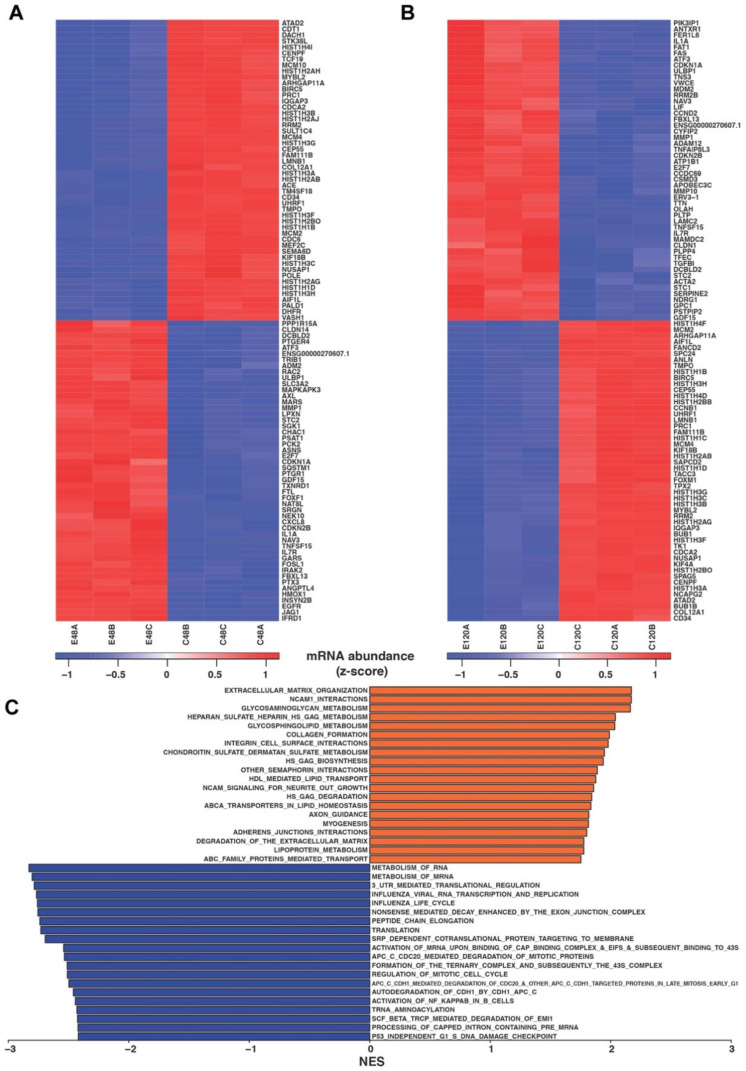
RNA-Seq analysis of coELTD1-expressing HUVECs. (**A**) Differential gene expression of top 50 upregulated genes and downregulated genes at 48 h (|log_2_FC| > 1 and FDR adjusted *p* value < 0.05). (**B**) Differential gene expression of top 50 upregulated genes and downregulated genes at 120 h (|log_2_FC| > 1 and FDR adjusted *p* value < 0.05). (**C**) Gene set enrichment analysis: Pre-ranked gene set enrichment analysis was performed using fgsea R package (v1.8.0) with REACTOME gene sets downloaded from MSig database [18]. Genes were ranked using: *sgn*(log_2_FC) × −log_10_ P. For our analysis, we applied a minimum gene set size of 10 genes and performed the analysis using 500 permutations.

To investigate the gene expression changes that occur after ELTD1 activation, RNA-Seq was performed immediately after coELTD1 expression (48 h) and after EndMT had occurred (120 h). Comparative transcriptomics at 48 h revealed 836 significantly differentially expressed genes (Top 50 most significant upregulated and down-regulated genes shown in Figure 5A and Table S2 contains full list ranked by fold change) and 683 significantly differentially expressed genes at 120 h (Figure 5B and Table S3). Of these genes, 484 were regulated at both 48 h and 120 h suggesting their importance in initiation and maintenance of the EndMT phenotype (Table S4). Validation of the gene changes by QPCR was performed on the 48 h and 120 h samples (Figure S3B,C). To characterise the genes which change differently at 120 h and 48 h, gene set enrichment analysis (GSEA) using the REACTOME pathway database was performed. GSEA revealed a significant downregulation (at 120 h) of pathways involved in RNA metabolism, cell cycle, DNA replication and translation which is consistent with the decrease in proliferation seen in the coELTD1 expressing cells. Consistent with an endothelial-myofibroblast transition there was an upregulation in many pathways associated with myofibroblast biology such as ECM organisation and integrin interactions, collagen formation and glycosaminoglycan and heparin sulphate metabolism (Figure 5C).

### 2.5. Factors Effecting Angiogenesis and EndMT to Myofibroblasts

**Figure 6:**
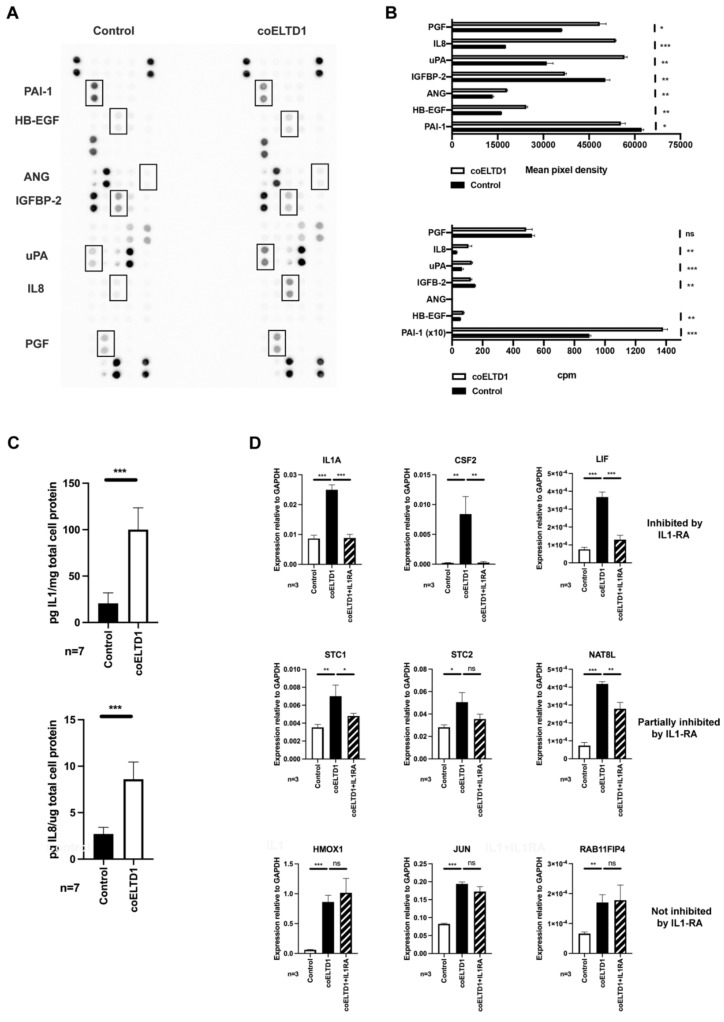
Pro-angiogenic secreted factors are released by coELTD1 expressing cells, including IL1. (**A**) ProteomeProfiler^TM^ Angiogenesis Array (R&D Systems) using supernatant collected from control and coELTD1 HUVEC collected at 120 h. (**B**) Quantification of the Angiogenesis array and comparison with RNA expression level. (**C**) Quantification of IL1 and IL8 secretion at 120 h using ELISA. (**D**) Expression of IL1 target genes upon IL1 treatment +/− IL1-receptor antagonist. * *p* < 0.05; ** *p* < 0.005; *** *p* < 0.0005.

coELTD1 expression results in a pro-angiogenic phenotype, therefore an angiogenesis array was performed on HUVEC supernatant collected 7 days post-infection to detect released factors that might be involved in this process. Five proteins were increased in the supernatant, HB-EGF, Angiogenin, uPA, IL8 and PGF and two were decreased, PAI-1 and IGFBP-2 (Figure 6A and quantification in Figure 6B), IL1α was not present on this array. RNA expression of *IL8*, *uPA*, *IGFBP*-2 and *HB-EGF* correlated with these changes. However, PGF expression was not significantly altered and PAI-1 expression decreased suggesting a different mechanism of regulation (Figure 6A,B).

coELTD1 expression also results in a form of EndMT. A number of pathways have been implicated in the activation of EMT. TGFB1 signalling has been the most intensively studied but other pathways such as IL6, IL8, FGF, Wnt, Notch, TNF and IL1 can promote a mesenchymal phenotype in a number of cell types [19,20]. Two of these, IL8 and IL1*α* were highly expressed at RNA level in coELTD1 cells at 48 and 120 h (Figure 5A and Tables S2 and S3). An ELISA was performed on HUVEC supernatants and this confirmed an increase in the secretion of these two factors in coELTD1 expressing cells (Figure 6C). IL1*α* increased endothelial sprouting, stress fibre formation and loss of cell-cell contact as seen with coELTD1 expression (Figure S4A,B). It also increased ELTD1 expression when treated for 72 h (Figure S4C,D). IL1*α*, IL8 and TGFB1 were added to endothelial cells but only IL1*α* could elicit the cell shape changes seen with coELTD1 expression (Figure S5A). To establish whether IL1*α* is solely responsible for the EndMT, IL1 receptor antagonist (IL1RA) was added to inhibit this pathway. IL1RA inhibited the expression of genes known to be regulated by IL1*α* (*CSF2* and *LIF*) however it did not inhibit EndMT induced by coELTD1 expression (Figure 6D and Figure S5B). Certain genes were partially inhibited by IL1RA (*STC1*, *STC2* and *NAT8L*) suggesting co-operation between ELTD1 and IL1*α* induced pathways. Other genes were unaffected and may represent ELTD1 direct targets such as *HMOX1*, *JUN* and *RAB11FIP4* (Figure 6D).

### 2.6. In Vivo Effects of ELTD1 Expression

**Figure 7:**
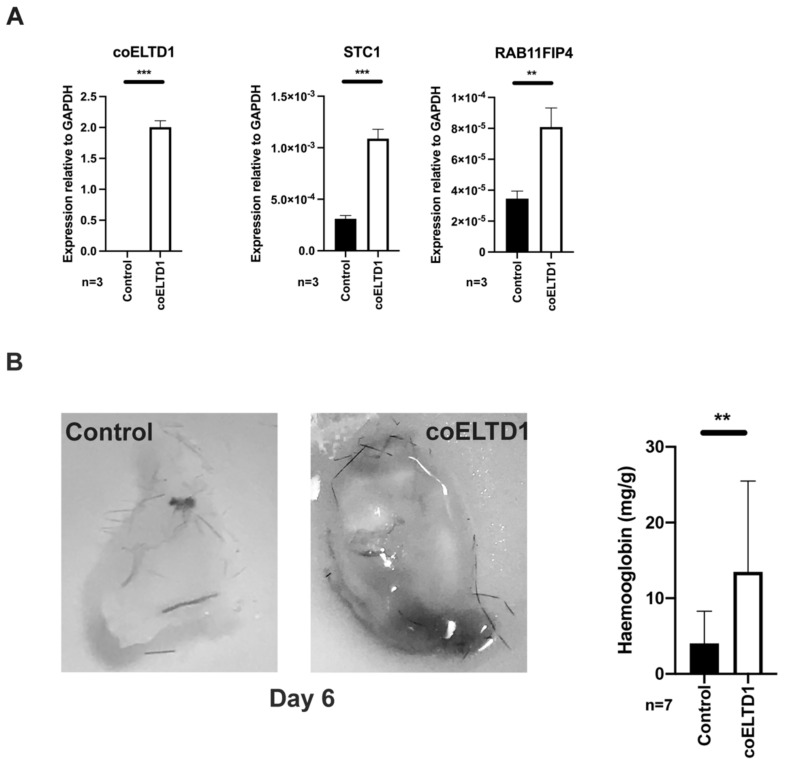
HUVEC expressing coELTD1 increase angiogenesis in vivo. HUVEC were infected with control or coELTD1 lentivirus and embedded in Matrigel^TM^ at 48 h. (**A**) Confirmation of *ELTD1* expression was done by QPCR along with genes that were shown to be up-regulated at 48 h by RNA-Seq. Matrigel^TM^ plugs were harvested and photographed before extraction and haemoglobin quantification at 6 days (**B**) post injection. ** *p* < 0.005; *** *p* < 0.0005.

To visualise the pro-angiogenic effects of coELTD1 expression in vivo, HUVEC were infected with coELTD1 expressing or control lentivirus and 48 h later they were injected into mice in Matrigel^TM^. coELTD1 expression was confirmed by QPCR along with the expression of genes which were identified during RNAseq analysis (Figure 7A).

Six days later the plugs were harvested and increased blood vessel formation was visualised in coELTD1 plugs. Quantification of the number of blood vessels was estimated indirectly by measuring haemoglobin levels in the plugs (Figure 7B).

## 3. Discussion

There are three types of EMT depending on its role; Type I is involved in embryogenesis and organ development, Type II occurs during wound healing and is associated with inflammation and fibrosis and Type III is involved in malignancy and metastasis [21]. Endothelial-mesenchymal transition (EndMT) occurs during heart development [22] and Eltd1 and Gpr116 have been reported to function together to regulate this process, with loss of these two proteins in mice leading to malformation of the aortic arch as well the development of a thrombotic microangiopathy [23]. We have also previously shown that eltd1 is important in blood vessel development in zebrafish with eltd1 silencing causing severe vascular defects [8].

EndMT has also been described in Type II EMT and results in loss of endothelial cell-cell junctions and a decreased expression of endothelial markers such as CD31 and CD34 [20]. After vessel damage there are three phases of the healing process; the inflammatory phase, the proliferation phase and the remodelling phase [24]. Inflammatory cytokines released during these processes attract endothelial cells, where they engage in angiogenesis, repair vessels and deliver oxygen and nutrients to the healing wound. They can also transform into myofibroblasts via EndMT [25] but their role in vessel repair remains unclear. EndMT was first described during embryonic heart development via TGF-β signalling [26]. In a porcine model, damage to the coronary artery resulted in the formation of vascular myofibroblasts.

Like fibroblast derived myofibroblasts, these non-muscle cells express αSMA and deposit collagen [27]. As the vessels are normally covered in smooth actin containing muscle cells, the myofibroblasts derived from endothelial cells could easily be overlooked and their role in vessel repair dismissed. Eltd1 has been implicated in cardiac hypertrophy, in mouse knockout models [28]. Lack of Eltd1 resulted in faulty cardiomyocytic remodelling produced by the cardiac pressure overload and a rise in myocardial fibrosis. Myofibroblasts not only express αSMA, they also have mature focal adhesions, form stress fibres and secrete ECM [29]. ELTD1-expressing cells possess all of these features and GSEA analysis of the RNA-Seq further confirms this phenotype with a number of pathways upregulated that are characteristic of myofibroblasts, such as ECM organisation and integrin interactions. As well as secreting collagen, they have a 14-fold increase in RNA expression of laminin β4 (LAMB4) at 48 h post infection. This and other myofibroblast derived ECM proteins may be involved in adhering ELTD1 to culture dish surfaces and its resulting deposition in cell tracks. Similar fibroblast derived tracks are thought to be involved in cancer invasion with tumour cells migrating behind invasive stromal fibroblasts [30].

Myofibroblasts exert contractile force in wounds [25] which has been reported to involve αSMA [31]. However wounds can still heal in SMA knock-out mice therefore other proteins may be involved [32]. RNA-Seq data showed increased expression of Titin (TTN) at 48 and 120 h. TTN is expressed in striated muscle and it provides tension in myofibrils as well as having sensing and signalling functions [33]. Expression of this protein may play an important role in endothelial derived myofibroblast function.

The GSEA also revealed a reduction in cell cycle and DNA replication which explains the reduction in proliferation seen in ELTD1 expressing cells. Myofibroblasts are only transiently present in acute wounds where they are removed by apoptosis or senescence, therefore a reduction in growth would be expected. When they persist they can cause scarring, organ fibrosis and excessive ECM deposition in cancer [34].

EndMT has been reported in a variety of pathologic settings including cancer [35], chronic pulmonary hypotension [36], hypertrophic cardiomyopathy [37] and cardiac fibrosis [26,35]. Tumours have been described as “a wound that never heals” and there are many similarities between the development of tumour stroma and the wound healing process [38]. The vast majority of fibroblasts in breast cancer have an activated phenotype [39] with a similar spindle-shaped morphology to myofibroblasts [40]. These have been termed cancer-associated fibroblasts (CAFs) and in certain tumours 40% have been reported to derive from endothelial cells [35]. CAFs secrete various cytokines, chemokines, growth factors and MMPs to degrade the ECM proteins and contribute to tumour proliferation, invasion, metastasis and angiogenesis [41].

The pro-angiogenic effects of coELTD1 expressing cells were confirmed in vivo using a Matrigel^TM^ plug assay. The angiogenesis array revealed an increase in a number of pro-angiogenic factors that may be involved in this effect such as HB-EGF [42], PGF [43] and uPA [44]. PAI-1, the inhibitor of uPA was decreased in the supernatant as was IGFBP-2. IGFB-2 is cleaved by PAPPA to release the pro-angiogenic factor IGF-1 [45]. *PAPPA* RNA expression was increased over 2-fold at 48 and 120 h and may be responsible for the decrease in IGFB-2 levels. RNA-Seq of ELTD1 expressing cells revealed a significant increase in *MMP1* and *MMP10* at both 48 h and 120 h, both of these proteases are present in cardiac myofibroblasts [46]. *VEGFA* RNA expression was increased 2-fold at 48 h, but the main factors released by coELTD1-expressing cells are involved in the immune response. CXCL8 had the largest increase at RNA level (8.9-fold at 48 h and 3.5-fold at 120 h) with a 3-fold increase in secretion as measured by ELISA and angiogenesis array. CXCL8 released from endothelial cells attracts neutrophils to the site of injury but it has also been implicated in fibrosis, angiogenesis and tumorigenesis [47].

IL1α has the next highest increase in RNA expression (5.3-fold at 48 h and 2.5-fold at 120 h) and a 5-fold increase in secretion measured by ELISA. IL1 is not only involved in the immune response, it is also pro-angiogenic and can help drive tumour growth and invasiveness [48]. Although IL1α is released by ELTD1 expressing cells and can induce EndMT it was not responsible for the EndMT induced by coELTD1. IL1 is known to engage in cross talk with pro-angiogenic molecules such as VEGF, with the mRNA expression profile of VEGF and IL1 treated HUVEC sharing 63% homology [49,50]. This may be another example of such cross talk as many of the gene expression changes seen in coELTD1 cells are seen in IL1 treated cells [51]. Other chemokines that were significantly increased at the RNA level are *CXCL1*, *CXCL3* and *CXCL5*. These are all pro-angiogenic and have increased expression in different tumour types [52].

A number of other genes were upregulated at 48 and 120 h. Three of these were in the top 10 most highly expressed at 48 h; *STC1*, *NAT8L* and *OLAH*. Stanniocalcin-1 is a secreted glycoprotein hormone that has many functions including calcium homeostasis [53]. It promotes wound healing and reepithelization in damaged tissues, inhibits vessel leakage and regulates macrophage functions [54]. It is expressed in CAFs and has been implicated triple negative breast cancer metastasis [55] and pulmonary fibrosis [54]. NAT8L and OLAH have both been implicated in lipid metabolism [56,57], lipid transport and homeostasis pathways were also upregulated in the GSEA analysis of ELTD1 expressing cells suggesting that this type of energy metabolism is important in endothelial myofibroblast formation and function.

The data presented in this paper show that ELTD1 induces a Type II EndMT. As an adhesion GPCR it has a large ECD that is thought to be involved in cell-cell/cell-matrix adhesion. In the case of ELTD1, this domain may be important in sensing vessel integrity with vessel damage or permeability triggering activation and consequent EndMT. Activation of the receptor may help repair the leaky vessels such as those found in tumours allowing better perfusion and drug delivery. IL1 secretion form several sources in the tumour microenvironment may be responsible for the upregulation reported in tumour vessels. We have previously shown that ELTD1 levels are increased in the tumour vasculature sand this correlates with a good prognosis [8]. Angiogenesis and inflammation are both hallmarks of cancer and are interdependent processes than can drive tumorigenesis [58]. ELTD1 is involved in both of these pathways and is also of interest as a driver of myofibroblast formation which is being specifically targeted for fibrosis and cancer [59], ELTD1 is therefore an attractive therapeutic target for a number of pathologic conditions.

## 4. Materials and Methods

### 4.1. Cell Culture and Reagents

Human umbilical cord endothelial cells (HUVECs) and human microvascular cells (HMEC-1) were purchased from Lonza (Basel, Switzerland) and cultured in their EGM2 and EGM2-MV media respectively. IL8 and IL1a (R&D Systems, Minneapolis, MN, USA) were added to cells at 10 ng/mL. IL1-receptor antagonist (Sigma, St. Louis, MO, USA) was added at 1 μg/mL. The endogenous sequence of ELTD1 is poorly expressed therefore the cDNA was codon optimised using the JCat bioinformatics tool (http://www.jcat.de/) (accessed on 31 November 2017) without altering the amino acid coding sequence. Codon optimised ELTD1 (coELTD1) and HA-tagged coELTD1 were cloned into pLenti6.2V5DEST (ThermoFisher, Waltham, MA, USA) and the vector alone was used as a control. Virus was produced in 293T and concentrated by ultracentrifugation using standard techniques. The viruses were titred using blasticidin resistance and endothelial cells infected at MOI 5 for all experiments.

### 4.2. Cell Matrix Adhesion Assay

HMEC-1 cells were seeded at 1 × 10^4^ cells per well into the Millicoat^TM^ ECM Screening Kit (Merck, Kenilworth, NJ, USA) and the assay performed according to the manufacturer’s instructions.

### 4.3. Cell-Cell Adhesion Assay

HMEC-1 were seeded at 1.6 × 10^6^ cells into a 10 cm dish and 50,000 cells into a 96 well plate and left to adhere. The next day, 2 μM calcein AM (ThermoFisher, Waltham, MA, USA) was added to the cells in the 10 cm dish and incubated for 1 h at 37 ℃. The cells were harvested in PBS + 4 mM EDTA and washed in PBS. After counting, the cells were resuspended at 2.5 × 10^5^/mL and 200 μΛ added to the cells in the 96-well plate. Two hundred μL of the cell suspension was lysed in lysis buffer (20 mM Tris pH 7.5, 150 mM NaCl, 1 mM EGTA, 1 mM EDTA, 1% Triton X-100) to obtain a total fluorescence reading. After 1 h the plate was washed gently with warm PBS and cells were lysed in 200 μL of buffer. The fluorescence was measured using a FLUOstar optima microplate reader (BMG Labtech, Ortenberg, Germany) and expressed as a percentage of total fluorescence.

### 4.4. Migration Assay

Migration was assessed using the IncuCyte^®^ live cell analysis system (Sartorius, Göttingen, Germany). HUVECs were grown to confluence in a 24-well ImageLock plate and a scratch wound was made using the IncuCyte^®^ wound maker (Sartorius, Göttingen, Germany). Images were collected every hour until the wound had closed and the images were analysed using the Fiji open source platform [60].

### 4.5. Proliferation Assay

HUVEC proliferation was assessed over a 72 h time period using the CYQUANT^TM^ cell proliferation assay (ThermoFisher, Waltham, MA, USA) according to manufacturer’s instructions.

### 4.6. Hanging Drop Endothelial Sprouting Assay

Hanging drops were generated as previously described [61]. Drops were embedded in Matrigel^TM^ or 2.5 mg/mL fibrin (Sigma, St. Louis, MO, USA) and images acquired after 48 h using an AMG Evos XL Core digital microscope (Fisher Scientific, Waltham, MA, USA). The sprouting area was quantified using Fiji.

### 4.7. Tube Formation Assay

HUVECs were plated on top of 200 µL of Matrigel^TM^ at 5 × 10^4^ cells/well in a 24-well plate (BD Biosciences, San Jose, NJ, USA). Images were collected for 24 h using the IncuCyte^®^ live cell analysis system (Sartorius, Göttingen, Germany) and tube formation was analysed by counting the number of complete polygons.

### 4.8. QPCR Protocol

RNA was extracted using TRI Reagent^®^ (Sigma, St. Louis, MO, USA) according to manufacturer’s instructions and reverse transcription was performed using the High Capacity cDNA Archive Kit (Applied Biosystems, Waltham, MA, USA). Q-PCR reactions were set up using SensiMix^TM^ SYBR (Meridian Bioscience, Cincinnati, USA) with 20 ng of cDNA and 0.3 μM of each oligonucleotide The QPCRs were run in a RotorGene Q (QIAGEN, Hilden, Germany) The cycling conditions used were: 95 °C for 10 min followed by 40 cycles of 95 °C for 15 s and 60 °C for 60 s. A list of primers used are supplied in Table S1.

### 4.9. Western Blotting

Proteins were separated using SDS-PAGE using standard techniques. Antibodies were purchased from the following companies: ELTD1 (CL4164, Sigma, St. Louis, MO, USA); β-actin conjugated to HRP (Sigma, St. Louis, MO, USA), HA-Tag (6E2, Cell Signaling, Danvers, MA, USA). Secondary antibodies were purchased from DAKO (Glostrup, Denmark).

### 4.10. Immunohistochemistry (IHC) and Immunocytochemistry (ICC)

Confocal microscopy was performed using an LSM 880 Confocal Microscope (Zeiss, Jena, Germany) or a Zeiss Observer spinning disc confocal microscope using standard protocols. Hoescht 33342 (ThermoFisher, Waltham, MA, USA) was used to stain nuclei. Antibodies used: ELTD1 (‘in house’ mouse mAb raised to the extracellular EGF domains, clone name 97.1); αSMA (1A4, Sigma, St. Louis, MO, USA); Alexa Fluor^®^ 568 Phalloidin (ThermoFisher, Waltham, MA, USA), Alexa Fluor^®^ 488 HA-Tag (Cell Signaling, Danvers, MA, USA); VE-Cadherin (BV13, ThermoFisher, Waltham, MA, USA), paxillin (Y113, Abcam, Cambridge, UK).

### 4.11. Live Cell Imaging

HUVEC were infected with control or HA-tagged coELTD1 and incubated for 5 days until the cells changed adopted a larger, spindle-like morphology. The cells were then stained with Alexa Fluor^®^ 488 HA-Tag (Cell Signaling, Danvers, MA, USA) for 10 min and imaged every minute using a Zeiss Observer spinning disc confocal microscope.

### 4.12. Collagen Assay

Collagen detection in cell culture media was assessed using a Sirius Red collagen detection kit (cat. # 9062, Chondrex, Woodinville, WA, USA) according to the manufacturer instruction without prior precipitation. Briefly, samples were mixed with the Sirius Red solution at 1:1 ratio, vortexed and incubated for 1 h at room temperature. After centrifugation at 10,000 rpm for 3 min, the pellet was washed with the washing solution and centrifuged again at 10,000 rpm for 3 min. The resulting pellet was dissolved using extraction buffer, vortexed and transferred to a 96-well plate for a subsequent measurement of the OD at 540 nm. Media and empty wells alone served as a negative control to exclude any non-specific signal. The values were normalized to the respective cellular protein content.

### 4.13. ELISA

Cell culture media was harvested from HUVEC that had undergone EndMT for 48 h. IL1α and IL8 ELISAs were carried out as per manufacturer’s instructions (R&D Systems, Minneapolis, MN, USA)

### 4.14. Angiogenesis Array

Cell culture media was harvested from HUVECs that had undergone EndMT for 48 h. Total cell protein was estimated and the volume of media was adjusted to represent the same amount of protein per condition. The ProteomeProfiler^TM^ Angiogenesis Array (R&D Systems, Minneapolis, MN, USA) was carried out according to manufacturer’s instructions.

### 4.15. RNA-Seq

Purification of mRNA, generation of double stranded cDNA and library construction were performed using NEBNext Poly(A) mRNA Magnetic Isolation Module (E7490) and NEBNext Ultra II Directional RNA Library Prep Kit (E7760L) from Illumina (San Diego, CA, USA). Sequencing was performed on an Illumina NovaSeq6000 as 150 bp paired end (PolyA RNA-seq). This generated 72 to 90.6 million reads per sample with FastQC used to evaluate the library quality. Paired-end reads (151 base pair long) were aligned to the human reference genome GRCh38, using STAR 2.5.1b [62] with quantMode GeneCounts and twopassMode basic alignment settings. Details of RNA-Seq analysis can be found in the Supplementary Methods.

### 4.16. In Vivo Matrigel^TM^ Plug Assay

All procedures were carried out in accordance with Home Office licence 30/3197. 400 μL of Matrigel^TM^ containing 4 × 10^4^ HUVEC infected with control or coELTD1 virus were subcutaneous injected into the flank of female wild-type B57BL.6 mice (aged 5–6 weeks; Charles River, Wilmington, MA, USA). Matrigel^TM^ was removed at day 6 then weighed and photographed. Half of the plug was homogenized in 0.1% Brij-35 lysis buffer and the haemoglobin levels were measured using Drabkin’s reagent as per manufacturer’s instructions (Sigma, St. Louis, MO, USA).

### 4.17. Statistical Analysis

Prism 8 (Graphpad Software, San Diego, CA, USA) was used to analyse the results. All data are represented as mean +/− standard deviation (SD). Student’s *t*-test was used to compare two unpaired groups. Significance is denoted as: * *p* ≤ 0.05, ** *p* ≤ 0.01, *** *p* ≤ 0.001

## Data Availability

RNA-Seq data will be publicly available through EBI ENA repository under accession number: PRJEB36900.

## Supplementary Materials

The following are available online [63,64,65] at https://www.mdpi.com/article/10.3390/ijms222011293/s1.
