# Supplementary material for: ELTD1 Activation Induces an Endothelial-EMT Transition to a Myofibroblast Phenotype
Source: Int J Mol Sci. 2021 Oct 19;22(20):11293. doi: 10.3390/ijms222011293 (PMC8539764; doi:10.3390/ijms222011293)
Supplement: Supplementary file 1 [file ijms-22-11293-s001.zip › ijms-1373613-supplementary.pdf]

SUPPLEMENTAL MATERIAL

ELTD1 Activation Induces an Endothelial-EMT Transition to a Myofibroblast Phenotype

Helen Sheldon [1,*], John Alexander [2], Esther Bridges [1], Lucia Moreira [3], Svetlana Reilly [3], Koon Hwee Ang [1],
Dian Wang [4], Salwa Lin [1], Syed Haider [2], Alison H. Banham [4] and Adrian L. Harris [1,*]

Supplementary methods.

RNA-Seq analysis

Annotation file used for feature quantifications was downloaded from GENCODE (v22) in GTF file format. Post alignment quality control was performed using RseQC [63]. In order to verify that our samples were codon-optimized for ELTD1, we aligned our samples using coELTD1 as reference. The codon-optimized variants were then visually inspected using Integrative Genome Viewer [64] (see Supplementary Figure 3A). Visualisations were created in R. All analyses were performed in R statistical programming environment v3.5.0. Differential mRNA abundance analysis was performed using edgeR package [65] in R using the model *~0 + group.* Genes with low expression were filtered out by retaining genes with count-per-million (CPM) counts > 1 in at least two samples. Samples were normalized using edgeR's TMM (trimmed mean of M-values) method and differential expression was determined using quasi-likelihood (QL) F-test. Results were further annotated using ENSEMBL gene annotations from R package org.Hs.eg.db (v3.7.0) (www.bioconductor.org). Genes with $|log_2FC| > 1$ and FDR adjusted P value < 0.05 were considered statistically significant. Data is publicly available through EBI ENA repository under accession number: PRJEB36900

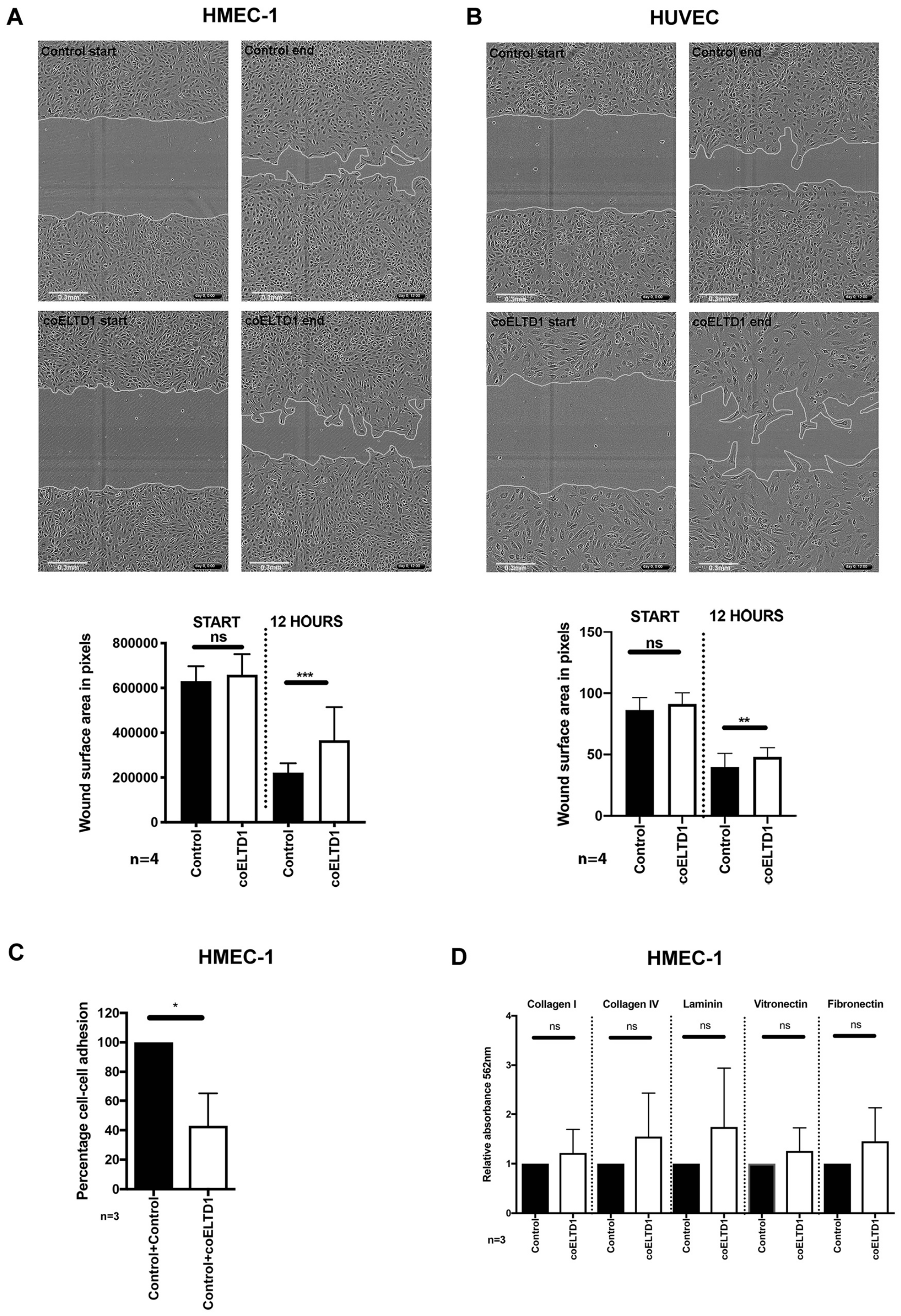

Figure S1: coELTD1 expression in HMEC-1 and HUVEC results in reduced migration and cell-cell adhesion. (A) HMEC-1 scratch wound migration assay with quantification. (B) HUVEC scratch wound migration assay with quantification. Images were taken using the IncuCyte® live cell analysis system (Sartorius) at ×10 magnification and wound closure analyzed using Fiji. (C) Cell-cell adhesion assay of control and coELTD1 expressing HMEC-1 cells. (D) ECM adhesion assay of control and coELTD1 expressing HMEC-1 cells.

Supplementary Figure 2 ELTD1 localisation in control and overexpressing cells

A

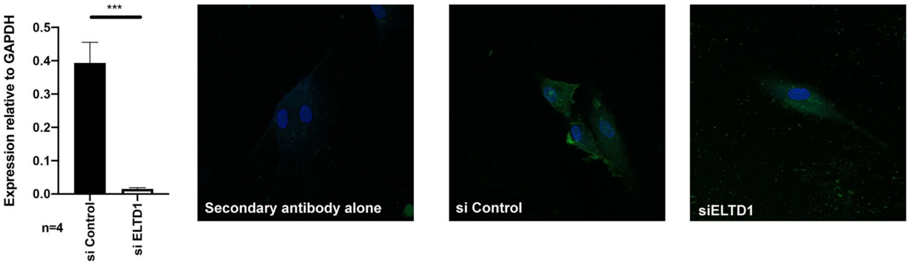

B

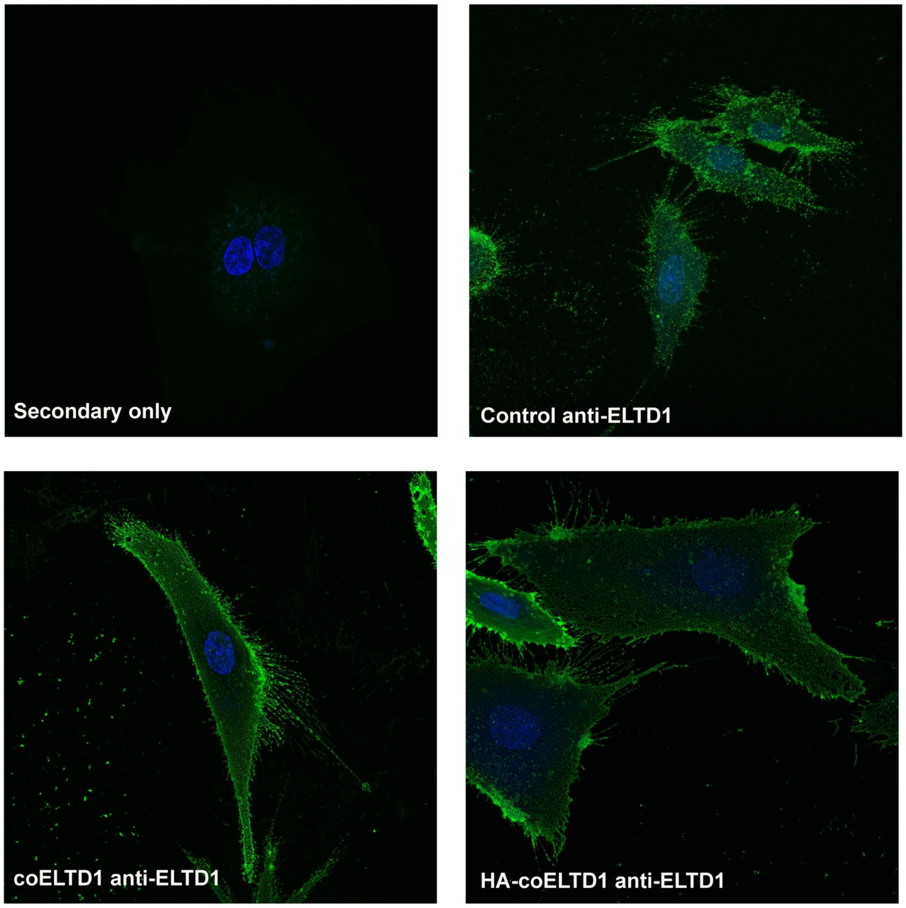

Figure S2: Increased expression and HA-tagging does not mislocalise ELTD1 (A) Silencing of ELTD1 was confirmed by QPCR and knock-down cells were used to confirm the specificity of the anti-ELTD1 antibody used for staining. (B) Fluorescent microscopy of HUVEC cells with secondary only antibody as background control compared to endogenous, coELTD1 and HA-tagged coELTD1 expression. Images were taken on a Zeiss LSM 880 Confocal Microscope (Zeiss) at ×40 magnification

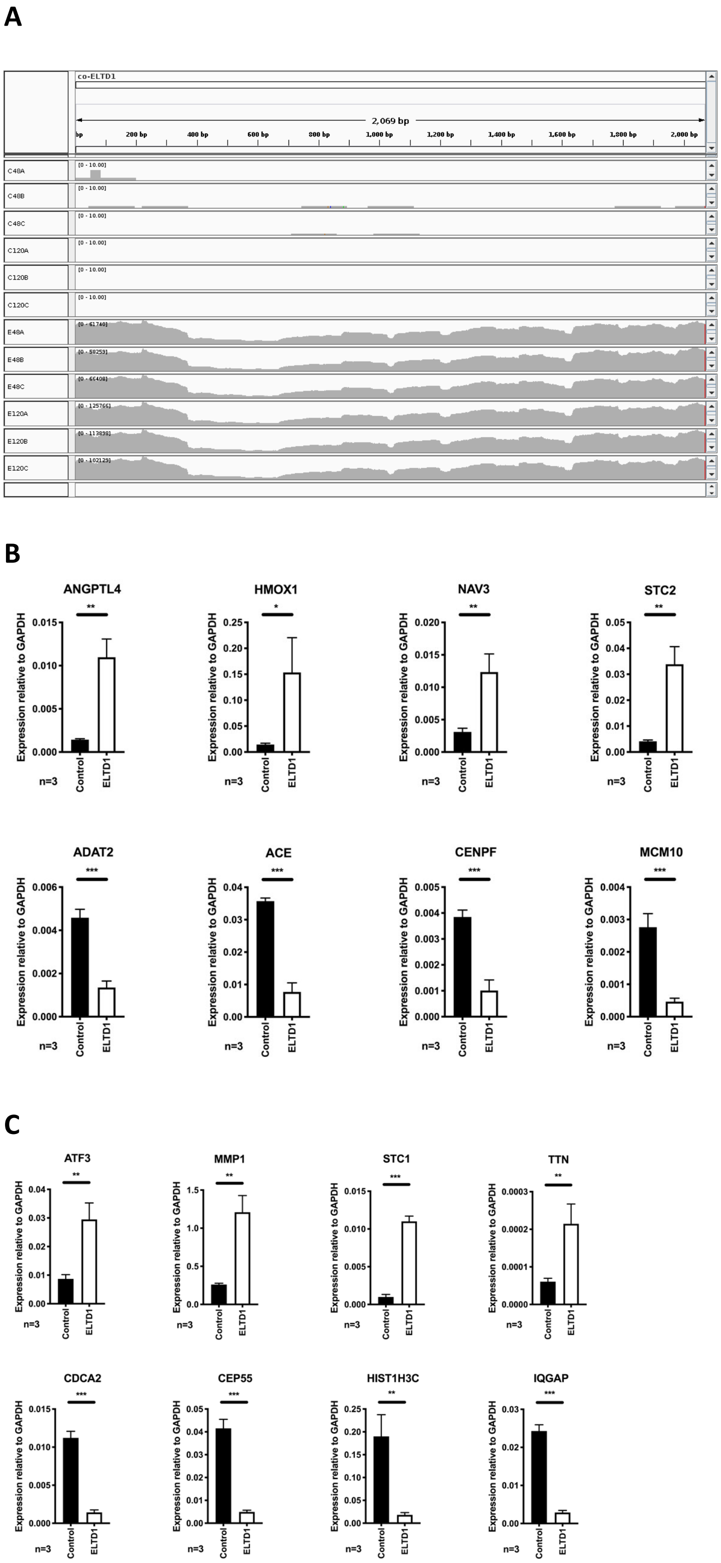

Figure S3: RNA-Seq validation. (A) Verification of coELTD1 expression in sequenced samples by alignment. (B) QPCR validation of up-regulated and down-regulated genes in 48hr samples and (C) 120hr samples.

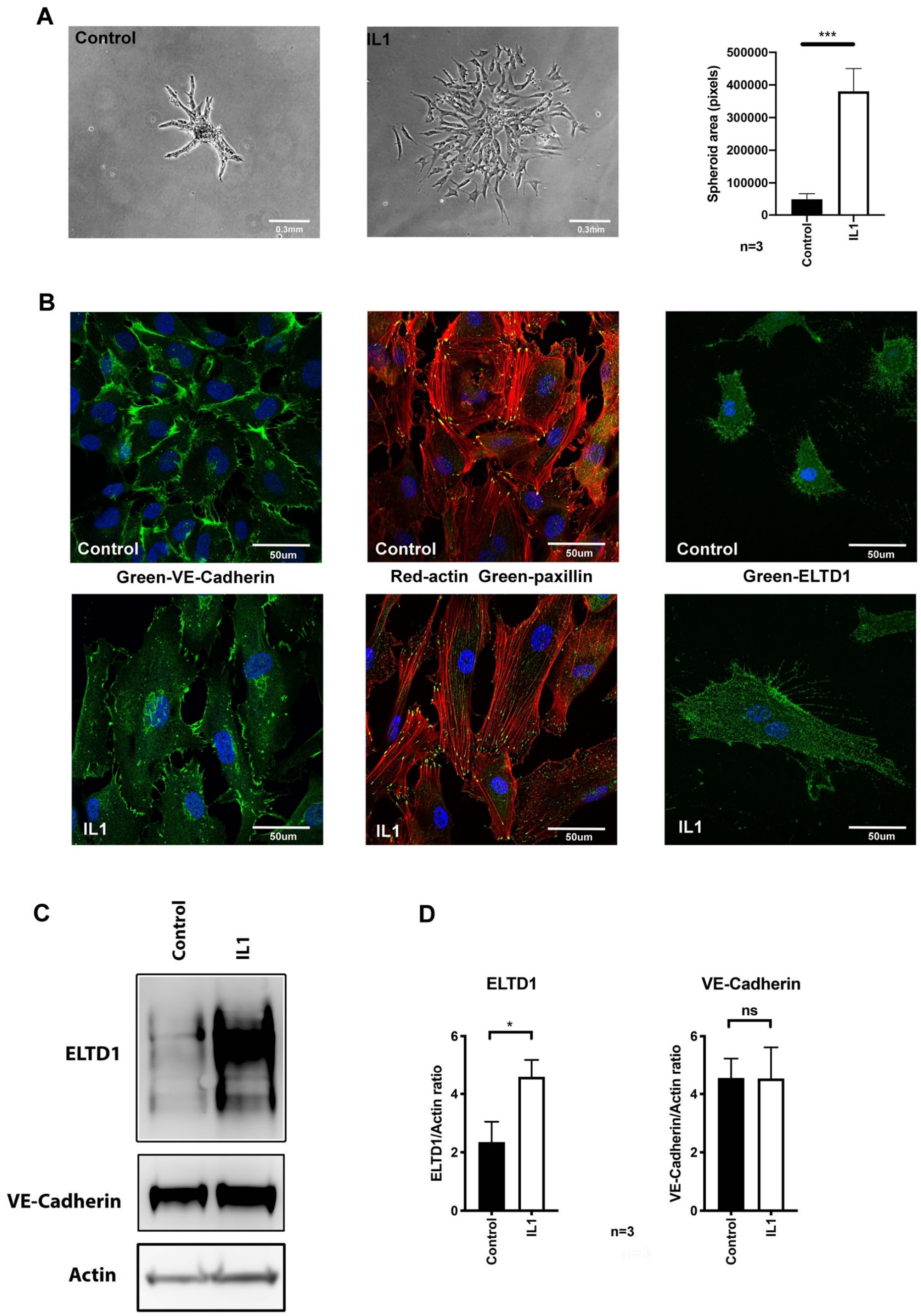

Figure S4: IL1 induces EndMT and ELTD1 expression. (A) Sprouting assay in fibrin gel of HUVEC treated with 10ng/ml IL1 with quantification of spheroid area. Images were taken on an AMG Evos XL Core digital microscope (Fisher Scientific) at 10x magnification. (B) Fluorescent microscopy of IL1 treated HUVEC using, actin (phalloidn), anti-paxillin and an ELTD1 antibody against the ECD. Images were taken on a Zeiss LSM 880 Confocal Microscope (Zeiss) at ×40 magnification. (C) Western blot of ELTD1 expression 72hrs after IL1 treatment with (D) quantification of protein expression compared to VE-Cadherin

A

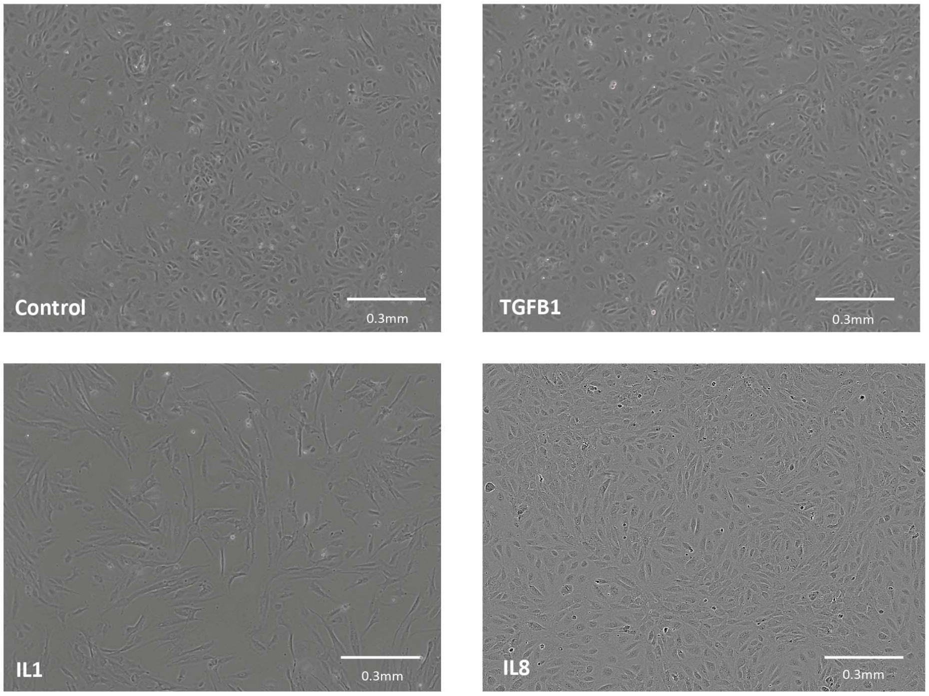

B

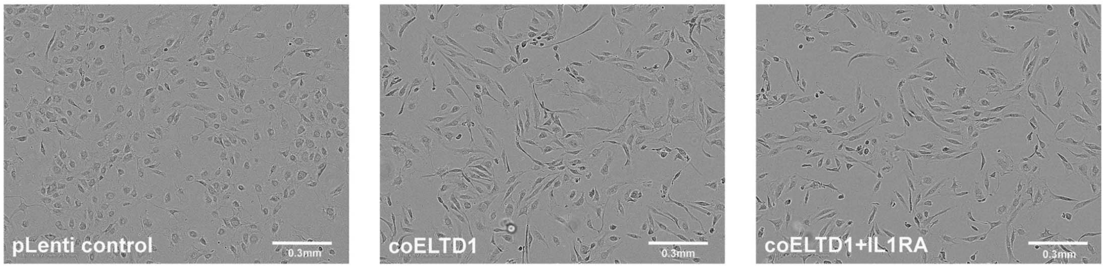

Figure S5: IL1 receptor agonist IL1-RA can not inhibit the EndMT induced by coELTD1 (A) Visualisation of HUVEC after treatment with 10ng/ml TGFB1, IL1 and IL8 (B) Visualization of EndMT after coELTD1 expression +/- IL1-receptor antagonist. Images were taken using the IncuCyte® live cell analysis system (Sartorius) at ×10 magnification.

Supplementary video legends.

Control Video S1: Appearance of HUVEC infected with control virus. Images were taken every 2hrs using the IncuCyte® live cell analysis system (Sartorius) at ×10 magnification.

coELTD1 Video S2: Appearance of HUVEC infected with coELTD1 virus. Images were taken every 2hrs using the IncuCyte® live cell analysis system (Sartorius) at ×10 magnification.

HA-tagged coELTD1 Video S3: Live Fluorescent microscopy of HUVEC infected with HA-tagged coELTD1 virus. Images were taken every 15 seconds using the Zeiss Observer spinning disc confocal microscope at ×63 magnification.
